# Berberine attenuate staphylococcal enterotoxin B-mediated acute liver injury via regulating HDAC expression

**DOI:** 10.1186/s13568-018-0684-2

**Published:** 2018-10-01

**Authors:** Jiying Du, Xiaohua Ding, Xiaoqin Zhang, Xinyu Zhao, Huidong Shan, Fanping Wang

**Affiliations:** 1 Institute of Inspection and Imaging, Sanquan Medical College, Xinxiang Medical University, Xinxiang, 453003 Henan China; 2 School of Medical Examination, Xinxiang Medical University, Xinxiang, 453003 Henan China; 3 Blood Immunology, School of Medical Examination, Xinxiang Medical University, No. 601, Jinsui Avenue, Xinxiang, 453003 Henan China

**Keywords:** BBR, SEB, HDAC, Splenocytes, Acute liver injury

## Abstract

Staphylococcal enterotoxin B (SEB) has been documented to be implicated in the pathogenesis of liver injury in the experimental models of hepatitis. However, the underlying mechanism of SEB-induced acute liver injury (ALI) remains to be further explored. In our study, we explored the therapeutic effectiveness of berberine (BBR), a natural isoquinoline alkaloid, in the SEB-induced ALI. In our study, we found that injection of SEB into d-galactosamine (d-gal)-sensitized mice induced ALI, as demonstrated by an increase of levels of alanine aminotransferase and aspartate aminotransferase, massive infiltration of immune cells into the liver, and pro-inflammatory cytokine release. However, intragastric administration of BBR attenuated SEB-induced ALI in mice. Meanwhile, we discovered that BBR treatment suppressed activation of splenocytes and pro-inflammatory cytokine release in SEB-stimulated splenocytes. Moreover, mechanistic analyses demonstrated that BBR was effective at inhibiting the expression of class I HDAC, but not class II, in SEB-stimulated splenocytes. Furthermore, trichostatin A, a standard HDAC inhibitor, alleviated activation of splenocytes and pro-inflammatory cytokine release in SEB-stimulated splenocytes. Taken together, we inferred from these results that BBR attenuated SEB-mediated ALI through repressing the class I HDAC enzyme, suggesting that BBR may constitute a novel therapeutic modality to prevent SEB-mediated inflammation and ALI.

## Introduction

*Staphylococcus aureus* (*S. aureus*), a ubiquitous Gram-positive opportunistic pathogen that can be found in 20% of the general human population, has emerged as a major cause of nosocomial infections and community acquired diseases (Pinchuk et al. 2010). Its pathogenicity can be attributed to a number of virulence factors including staphylococcal enterotoxin B (SEB) (Foster 2004). Commonly referred to as a superantigen, SEB directly binds to the non-polymorphic regions of the major histocompatibility complex class II (MHC II) on antigen-presenting cells (APCs) and the variable region of the β-chain of the T-cell receptor (TCR) (Kozono et al. 1995). The consequent activation of a substantial number of T lymphocytes (approximately 30–40%) leads to the uncontrolled production of inflammatory cytokines, such as interleukin (IL)-2, IL-6, tumor necrosis factor-α (TNF-α), and interferon-γ (IFN-γ), producing an adverse inflammatory response (Baker and Acharya 2004). The consequence of SEB exposure is associated with a wide range of human diseases ranging from mild food poisoning-like symptoms to more severe and potential fatal conditions, such as lethal toxic shock (Larkin et al. 2009; Uchakina et al. 2013). Notably, SEB has been documented to be implicated in the pathogenesis of liver injury in the experimental models of hepatitis (Yasuda et al. 2002).

Berberine (BBR) is a natural isoquinoline alkaloid traditionally used for the treatment of hepatic disorders, which is extracted from fresh roots, rhizome, and stem bark of numerous clinically important medicinal plants such as *Berberis aquifolium* or *Coptis chinensis* (Zuo et al. 2006). Recently, ample evidence has suggested that BBR extracts have multiple beneficial pharmacological effects including anti-inflammation (Bae and Cheon 2016), anti-oxidant (Jung et al. 2009), anti-bacterial (Yu et al. 2005), immunoregulative (Kim et al. 2003), and hepatoprotective (Ye et al. 2009). Recently, extensive researches have also documented the inhibitory effects of BBR on chemically induced cytotoxicity and oxidative stress in the liver (Hwang et al. 2002; Zhang et al. 2008). For example, BBR was demonstrated to be effective in protecting the liver from acute carbon tetrachloride (CCl_4_)-induced injury (Domitrovic et al. 2011), and doxorubicin-induced acute hepatorenal toxicity in rats (Chen et al. 2016). Although the hepatoprotective effect of BBR has been characterized, the roles of BBR in SEB-induced acute liver injury (ALI) are still undefined.

Histone acetylation via histone deacetylases (HDACs) is an epigenetic modification which is associated with the transcriptional activation by modulating chromatin condensation. HDACs regulate transcriptional process through deacetylation of histone and non-histone proteins (Wu and Grunstein 2000). HDACs can be phylogenetically divided into four subclasses based on function and sequence homology, including class I (HDAC 1, 2, 3, and 8), class II (HDAC 4, 5, 6, 7, 9 and 10), class III (SIRT1-7), and class IV (HDAC11) (de Ruijter et al. 2003). Notably, recent studies have proofed that class I HDAC plays a critical role in promoting T cell activation and inflammatory response induced by SEB, while class II HDAC may attenuate this response (Busbee et al. 2014). The present study was designed to assess the protective effects of BBR against SEB-induced acute liver injury and the underlying mechanism.

## Materials and methods

### Mice

The female C57BL/6 mice (6–8 weeks old) used in these experiments were purchased from Shanghai Model Organisms Center, Inc (Shanghai, China) and housed under pathogen-free conditions in Laboratory Animal Center of Xinxiang Medical University. All animal experimental procedures were performed with the approval of the Xinxiang Medical University Animal Care and Use Committee.

### ALI mice model and treatment

To establish an in vivo SEB-induced ALI mouse model, SEB (BT202, Toxin Technologies, Sarasota, FL) dissolved in sterile PBS was injected intraperitoneally into age- and weight-matched female C57BL/6 mouse in a volume of 100 μl for a dose of 40 µg (Hegde et al. 2011). These mice were randomly divided into three groups (n = 10 per group): vehicle group, SEB + vehicle group, and SEB + BBR group. For the treatment group, these mice received an intragastric administration with BBR (Sigma-Aldrich, St. Louis, MO, USA) dissolved in PBS through oral gavage at 100 mg/kg body weight in a volume of 100 μl every other day for 5 days prior to SEB injection. The mice in vehicle group and SEB + vehicle group received an intragastric administration with 100 μl PBS every other day for 5 days prior to SEB injection. Mice were monitored daily and euthanized 24 h after SEB injection. Blood and serum were separated by centrifugation (4 °C, 3200×*g* for 20 min) and stored at − 20 °C before use. The liver tissue samples were collected for histological analyses.

### Biochemical and histological analyses

The serum levels of liver marker enzymes including alanine aminotransferase (ALT) and aspartate aminotransferase (AST) in serum were measured using commercially available diagnostic kits (Alanine aminotransferase assay kit; cat no. C009-2; Aspartate aminotransferase assay kit; cat no. C010-2; Nanjing Jiancheng Bio Co., Ltd., Nanjing, China). The collected liver tissues were fixed in 10% formalin, embedded in paraffin, and cut into 5 µm sections. Subsequently, the sections were deparaffinized in xylene, rehydrated in alcohol (100, 95, and 90%) and stained with hematoxylin–eosin (H&E). The stained sections were examined under a Nikon E600 light microscope (Nikon, Tokyo, Japan) at 40× magnification.

### Splenocytes isolation and detection

Splenocytes were prepared by aseptically removing the spleens from naive C57BL/6 mice. Spleens were homogenized into single-cell suspensions using a Stomacher 80 Biomaster blender (Seward, Davie, FL). The resulting suspension was centrifuged at 1600×*g* for 30 min and then subjected to red blood cell lysis (Sigma-Aldrich) according to the manufacturer’s instructions. The single splenocytes were collected and plated in a 96-well plate in RPMI 1640 media supplemented with heat inactivated 10% fetal bovine serum, 10 mM l-glutamine, 10 mM HEPES, 50 µM β-mercaptoethanol, and 100 µg/ml penicillin/streptomycin at a density of 1 × 10^6^ cells/well at 37 °C. Splenocytes were stimulated with PBS or 1 µg/ml SEB, followed by treatment with BBR (1, 2, 4, or 8 μM) or 100 nM tichostatin A (TSA) for 24 h. To assess activation, splenocytes collected from in vitro culture were stained with anti-mouse CD69 antibody (Biolegend, San Diego, CA, USA). Flow cytometry analysis was conducted in splenocytes using a FACS Calibur flow cytometer (BD Biosciences, San Jose, CA, USA) and the number of splenocytes was analyzed by Cell Quest Pro (BD Biosciences).

### Cell preparation and flow cytometry

Liver infiltrating mononuclear cells were separated and counted 24 h after SEB administration by Percoll density separation, as described (Hegde et al. 2011). To determine the phenotypical characteristics of the liver infiltrating cells and splenocytes isolated as above, cells were stained with the following fluorescent-conjugated antibodies: fluorescein isothiocyanate (FITC)-conjugated anti-CD8 (clone: 53-6.7), anti-CD3 (clone: 145.2 C11), phycoerythrin (PE)-conjugated anti-CD4 (clone: GK 1.5), anti NK1.1 (clone: PK136), from Biolegend, and PE-conjugated anti-Vβ8 from Ebioscience (San Diego, CA, USA). Stained cells were analyzed using Beckman Coulter 500 Flow Cytometer (Indianapolis, IN).

### Cytokines analysis in culture supernatant and serum

Cell supernatants were collected from treated splenocytes after 24 h. The cytokine levels of IFN-γ, TNF-α, IL-6 and IL-2 in the isolated serum samples from mice or cell supernatants of the treated splenocytes were analyzed and quantified using individual enzyme-linked immunosorbent assay (ELISA) kits (Biolegend).

### Cytotoxicity assay

MTT assay was performed to evaluate BBR-induced cytotoxicity against splenocytes. Briefly, the treated splenocytes were plated into a 96-well plate at approximately 1 × 10^6^ cells per well at 37 °C and cultured with fresh RPMI 1640 media containing different doses of BBR (1, 2, 4, or 8 μM). Following incubation for 24 h, 20 μl of MTT solution (5 mg/mL, Sigma-Aldrich) was added into each well and incubated at 37 °C for another 4 h. Subsequently, the culture medium was removed and 150 μl of dimethyl sulfoxide (DMSO) was added to dissolve the formazan crystals. The absorbance at 490 nm was detected using a microplate reader (Bio-Rad, Hercules, CA, USA).

### HDAC activity

The HDAC activity was estimated using a colorimetric HDAC activity assay kit (BioVision Research Products, Mountain View, CA, USA). Briefly, proteins were extracted from treated splenocytes using RIPA lysis buffer (Beyotime Institute of Biotechnology, Shanghai, China) and quantified by BCA Protein Assay Kit (Pierce, Rockford, IL, USA). 100 μg of total extracts dissolved in a final volume of 85 μl ddH_2_O were added into the 96-well plates. Then, 10 μl of 10× HDAC assay buffer were added to each well, followed by 5 μl of the colorimetric substrate. After incubation at 37 °C for 1 h, 10 μl of lysine developer was added and incubated for 30 min at 37 °C to arrest the reaction. The absorbance was obtained by an ELISA plate reader at 405 nm.

### Quantitative RT-PCR (qRT-PCR) analysis

Total RNA from the treated splenocytes was extracted using TRIzol reagent (Qiagen, Hilden, Germany) and quantified using the Nanodrop 2000 spectrophotometer (Thermo Fisher Scientific, Waltham, MA, USA). For the detection of mRNA, 2 μg of total RNA was reversely transcribed into cDNA using PrimeScript RT reagent Kit (Bio-Rad, Hercules, CA, USA). Subsequently, RT-PCR was performed with SYBR Prime Script RT-PCR Kits (TaKaRa, Otsu, Shiga, Japan) on an ABI PRISM 7900 Real-Time system (Applied Biosystems, Foster City, CA, USA), with GAPDH as an endogenous control. The reaction protocol was as follows: denaturation at 95 °C for 10 min, followed by 40 cycles of denaturation 95 °C for 30 s, annealing at 60 °C for 1 min and extending at 72 °C for 30 s. The relative gene expression was calculated using the 2^−∆∆Ct^ method.

### Western blot

Total protein was extracted from the treated splenocytes using RIPA lysis buffer and protein concentration was detected by BCA Protein Assay Kit. Cellular extracts (20 µg per lane) were loaded onto 10% sodium dodecyl sulfate–polyacrylamide gel electrophoresis (SDS-PAGE) and transferred to a nitrocellulose membrane (Millipore, Billerica, MA, USA). The membranes were blocked with 5% non-fat dry milk in Tris-buffered saline for 1 h at room temperature and then incubated with the primary antibody against acetylated histone H3 lysine 9 (H3K9Ac) (1:500; Abcam, Cambridge, MA, USA) and histone H3 (1:1000; Cell Signaling Technology, Beverly, MA, USA) at 4 °C overnight. After washing with TBST for 3 times, the membranes were further incubated with the corresponding horseradish peroxidase (HRP)-conjugated secondary antibodies (1:1000; Abcam). Subsequently, signals were detected using an enhanced chemiluminescence detection kit (Pierce).

### Statistical analysis

All results were expressed as the mean ± standard deviation (SD). All statistical analyses were performed using SPSS version 17.0 (SPSS, Inc., Chicago, IL, USA) software with unpaired Student’s *t*-test or one-way analysis of variance (ANOVA). *P* values < 0.05 were considered to indicate a statistically significant difference.

## Results

### BBR attenuated SEB-induced ALI in mice

**Fig. 1:**
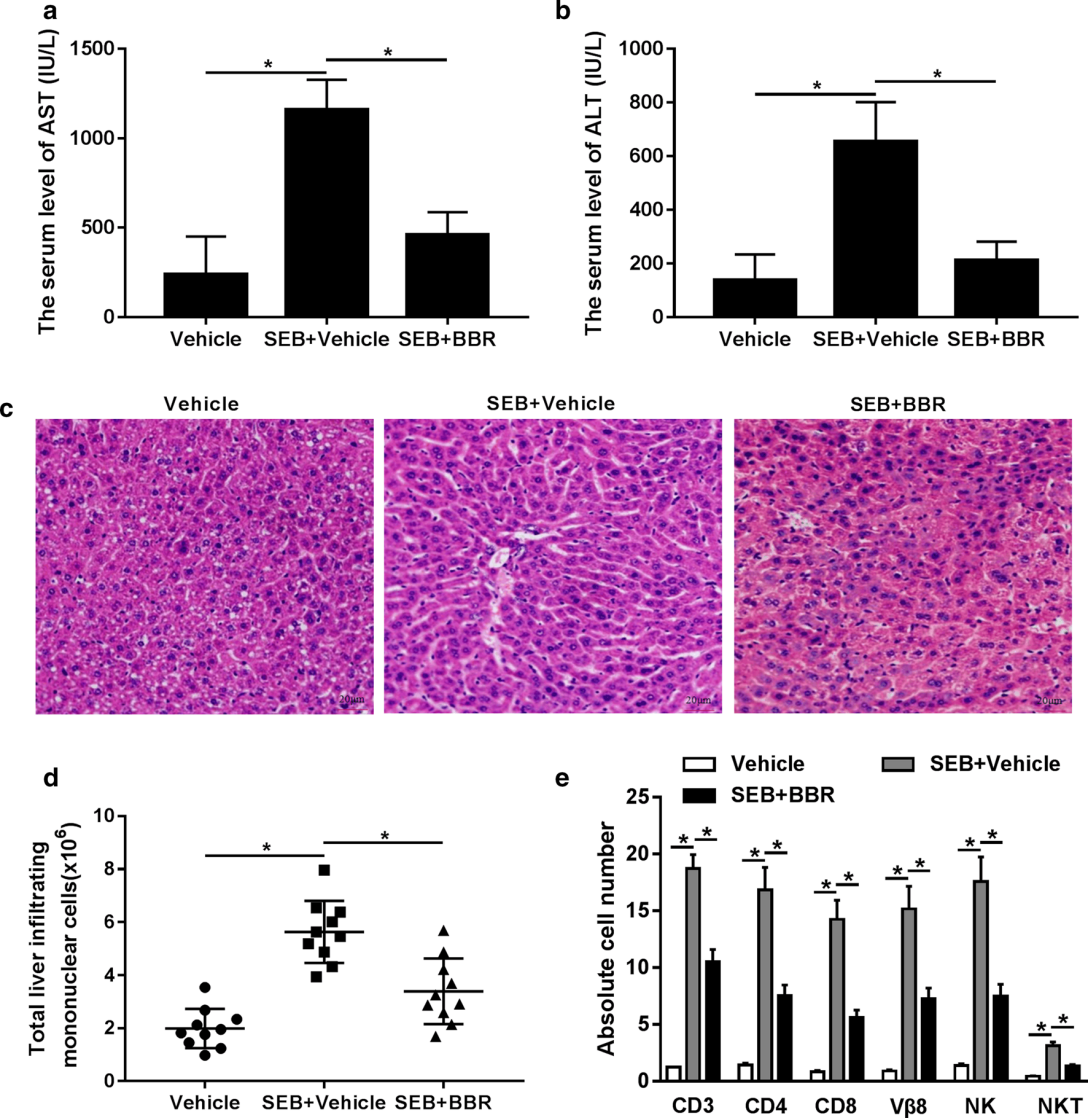
BBR attenuated SEB-induced ALI in mice. **a**, **b** The serum levels of ALT and AST were determined. **c** Histopathological examination of the liver tissues was conducted by HE staining. **d** Liver-infiltrating mononuclear cells were counted. **e** The liver infiltrating mononuclear cells were stained with monoclonal antibodies to determine the following subsets: T cells (CD3), T-helper cells (CD4), T-cytotoxic cells (CD8), natural killer cells (NK), and natural killer T-cells (NKT). *P < 0.05. SEB + Vehicle group versus Vehicle or SEB + BBR group, respectively

To address the effect of BBR on SEB-induced ALI, these mice were initially sensitized by giving them an intraperitoneal injection of 20 mg of d-galactosamine (d-gal) in 100 μl phosphate-buffered saline (PBS) 30 min prior to SEB injection, as previously described (Hegde et al. 2011). In addition, these mice received an intragastric administration with BBR prior to SEB stimulation. As shown in Fig. 1a, b, the serum levels of liver marker enzymes including AST and ALT were significantly elevated in d-gal-sensitized mice following SEB challenge compared with the Vehicle group, suggesting that SEB induced extensive ALI. However, BBR administration greatly undermined SEB-induced increase of AST and ALT levels in d-gal-sensitized mice. Intriguingly, BBR alone showed little effect on the levels of ALT and AST (data not shown). Histopathological examination of the liver tissues by HE staining showed that the liver tissues from mice sensitized with d-gal and treated with SEB exhibited remarkable amounts of infiltrating cells with respect to the liver tissues from mice sensitized with d-gal and treated with Vehicle, which was distinctly attenuated in the liver tissues from sensitized with d-gal and treated with SEB and BBR (Fig. 1c). Moreover, we found that the number of total liver infiltrating mononuclear cells isolated from the liver was dramatically enhanced by SEB challenge compared with Vehicle group, while cotreatment with BBR and SEB markedly reduced this number (Fig. 1d). The liver infiltrating mononuclear cells were further analyzed to identify the different immune subsets. We stained the cells with various fluorescein-conjugated antibodies and the results proved that SEB administration led to an evident increase in the total number of cells expressing CD3^+^ (T-cells), CD4^+^ (T-helper cells), CD8^+^ (cytotoxic T-cells), Vβ8^+^ NK^+^ (NK cells) and NK1.1^+^ CD3^+^ (NK T-cells) (Fig. 1e). However, BBR exposure apparently reduced the absolute cell numbers (Fig. 1e). Collectively, these data revealed that BBR attenuated SEB-induced ALI in mice.

### BBR alleviated SEB-induced inflammation in the liver

**Fig. 2:**
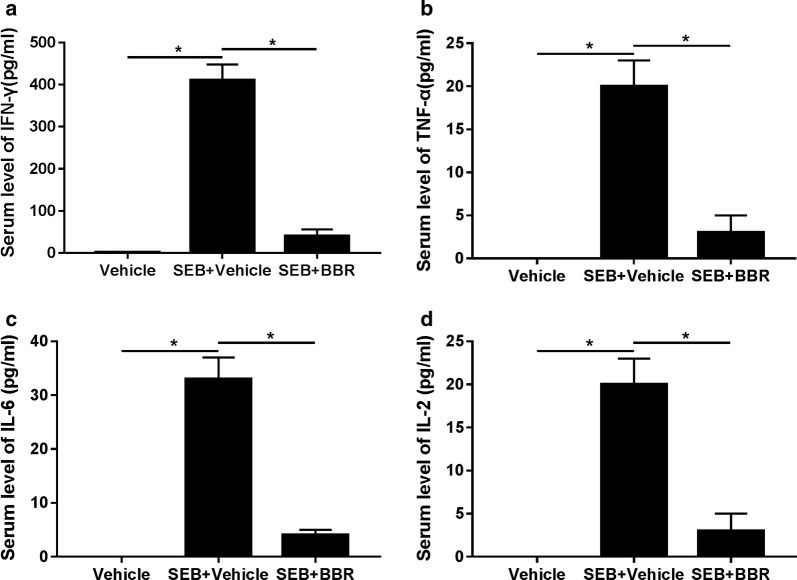
BBR alleviated SEB-induced inflammation in the liver. The levels of IFN-γ **a**, TNF-α **b**, IL-6 **c** and IL-2 **d** in the serum from mice sensitized with d-gal and treated with SEB or combined with BBR and were examined by ELISA. The level of IFN-γ, TNF-α, IL-6 and IL-2 in vehicle was 5.89 ± 1.22, 0.16 ± 0.02, 0.23 ± 0.02, 0.11 ± 0.01, respectively. *P < 0.05. SEB + Vehicle group versus Vehicle or SEB + BBR group, respectively

SEB-mediated inflammation triggered the rapid release of pro-inflammatory cytokines. To explore the effects of BBR on SEB-induced inflammation, we measured the levels of pro-inflammatory cytokines including IFN-γ, TNF-α, IL-6 and IL-2 in the serum from mice sensitized with d-gal and treated with SEB or combined with BBR. ELISA results presented that the serum levels of IFN-γ (Fig. 2a), TNF-α (Fig. 2b), IL-6 (Fig. 2c) and IL-2 (Fig. 2d) were all considerably increased after SEB exposure, while BBR treatment prominently reduced SEB-induced increase of these pro-inflammatory cytokine levels. The level of IFN-γ, TNF-α, IL-6 and IL-2 in vehicle was 5.89 ± 1.22, 0.16 ± 0.02, 0.23 ± 0.02, 0.11 ± 0.01, respectively. These results indicated that BBR alleviated SEB-induced inflammation in the liver.

### BBR suppressed activation of splenocytes and pro-inflammatory cytokine release in SEB-stimulated splenocytes

**Fig. 3:**
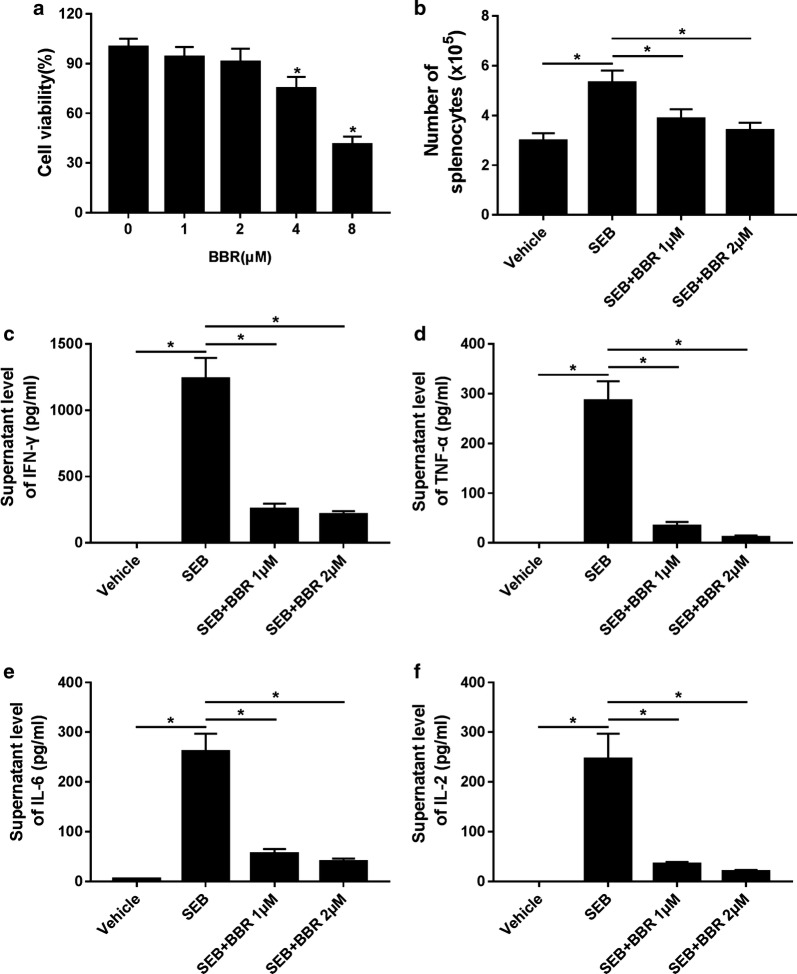
BBR suppressed SEB-induced activation of splenocytes and pro-inflammatory cytokine release in splenocytes. **a** BBR-induced cytotoxicity against splenocytes was assessed by MTT assay. **b** The number of the treated splenocytes was detected by flow cytometry analysis. The levels of IFN-γ **c**, TNF-α **d**, IL-6 **e** and IL-2 **f** in the supernatants of splenocytes were measured by ELISA. *P < 0.05. SEB group versus Vehicle or SEB + BBR group, respectively

BBR-induced cytotoxicity against splenocytes was assessed by MTT assay and the results showed that BBR at 4 or 8 μM led to a dramatic reduction of cell viability in splenocytes, while BBR at 1 or 2 μM exhibited no obvious influence on cell viability of splenocytes (Fig. 3a). Therefore, 1 or 2 μM BBR was chosen for subsequent experiments. Splenocytes were exposed to 1 or 2 μM SEB in the presence or absence of BBR for 24 h and the number of splenocytes after SEB treatment was remarkably elevated, but substantially declined following the addition of 1 or 2 μM BBR (Fig. 3b), suggesting that BBR suppressed SEB-induced activation of splenocytes. Furthermore, we analyzed the concentrations of pro-inflammatory cytokines in the supernatants from the aforementioned experiments by ELISA and the results revealed that BBR administration at 1 or 2 μM both effectively weakened SEB-induced increase of IFN-γ (Fig. 3c), TNF-α (Fig. 3d), IL-6 (Fig. 3e) and IL-2 (Fig. 3f) levels in the supernatants of splenocytes. Together, these findings indicated that BBR suppressed SEB-induced activation of splenocytes and pro-inflammatory cytokine release in splenocytes.

### BBR was effective at inhibiting the gene expression of class I HDAC in SEB-stimulated splenocytes

**Fig. 4:**
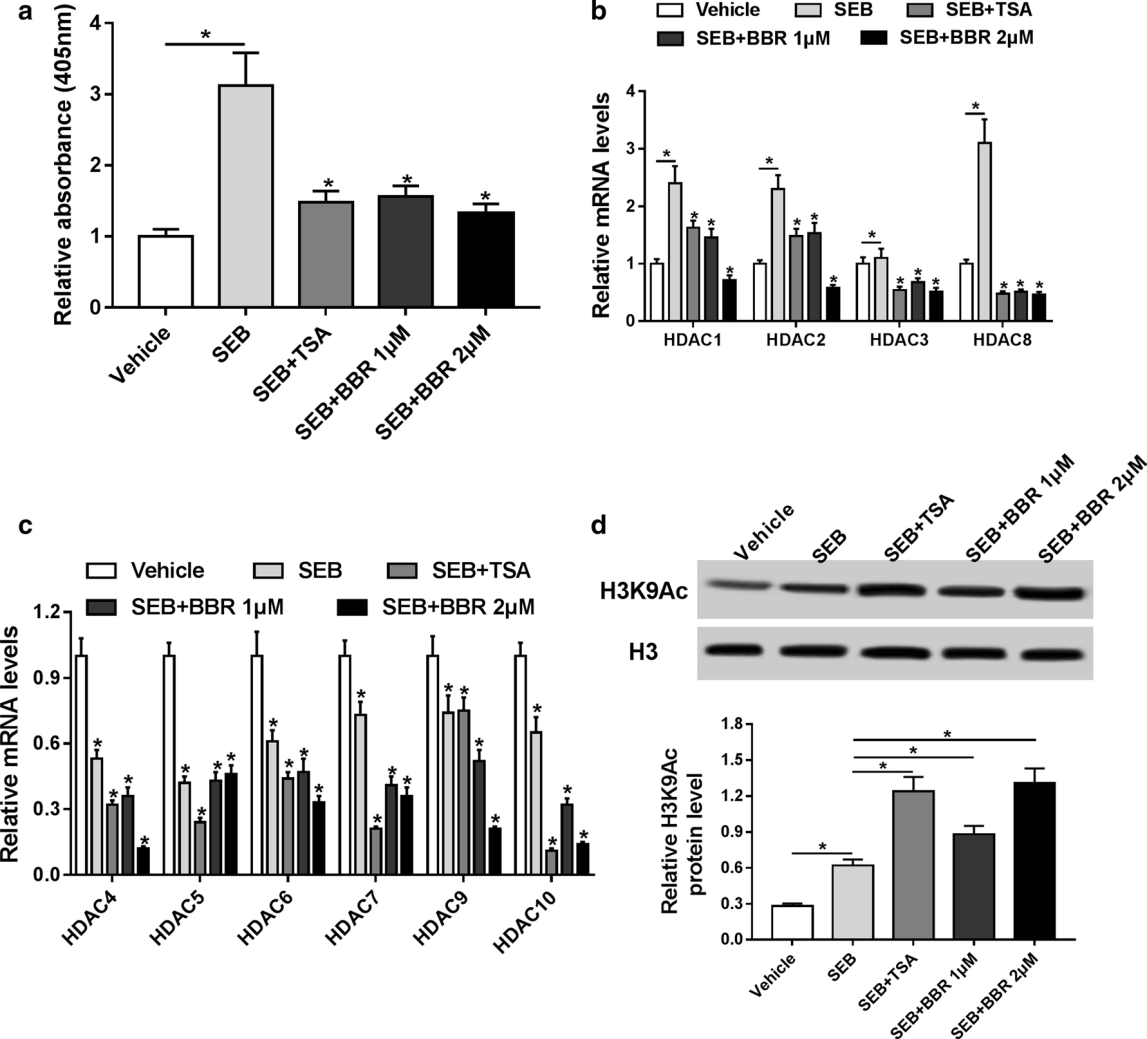
BBR was effective at downregulating class I HDAC after activation of SEB. Splenocytes were treated with SEB, in the absence or presence of 1 or 2 μM BBR or 100 nM TSA for 24 h. **a** The HDAC activity was investigated by colorimetric analysis. **b**, **c** The mRNA expression of class I and II HDAC enzymes was detected in the treated splenocytes by qRT-PCR analysis. **d** The protein levels of H3K9Ac were examined by western blot in the treated splenocytes. *P < 0.05. SEB group versus Vehicle or SEB + BBR or SEB + TSA group, respectively

To decipher the mechanism underlying the effects of BBR on SEB-induced ALI, we analyzed whether BBR could regulate the expression of HDAC in SEB-stimulated splenocytes. Splenocytes were treated with SEB, in the absence or presence of 1 or 2 μM BBR or 100 nM TSA, a standard HDAC inhibitor which selectively inhibits the class I and II HDAC enzymes. Colorimetric assay analysis demonstrated that the HDAC activity of SEB-stimulated splenocytes was notably increased versus Vehicle group, which was drastically abated by 1 or 2 μM BBR and TSA (Fig. 4a). We further examined the effects of BBR on the expressions of class I and II HDAC enzymes and qRT-PCR analyses showed that treatment with SEB significantly upregulated the expressions of class I HDAC (HDAC1, HDAC2, HDAC3, and HDAC8) and downregulated the expressions of class II HDAC (HDAC4, HDAC5, HDAC6, HDAC7, HDAC9, and HDAC10) in comparison to unstimulated cells (Fig. 4b, c). However, 1 or 2 μM BBR and TSA treatment both strikingly suppressed the expressions of class I HDAC in SEB-stimulated splenocytes. Moreover, western blot demonstrated that H3K9Ac level was dramatically increased in SEB-stimulated splenocytes compared to Vehicle group, which may be attributed to the decrease of class II HDAC enzyme (Fig. 4d). Notably, 1 or 2 μM BBR and TSA treatment led to a marked elevation of the protein level of H3K9Ac in SEB-stimulated splenocytes, suggesting that BBR was effective at downregulating class I HDAC after activation of SEB.

### TSA alleviated activation of splenocytes and pro-inflammatory cytokine release in SEB-stimulated splenocytes

**Fig. 5:**
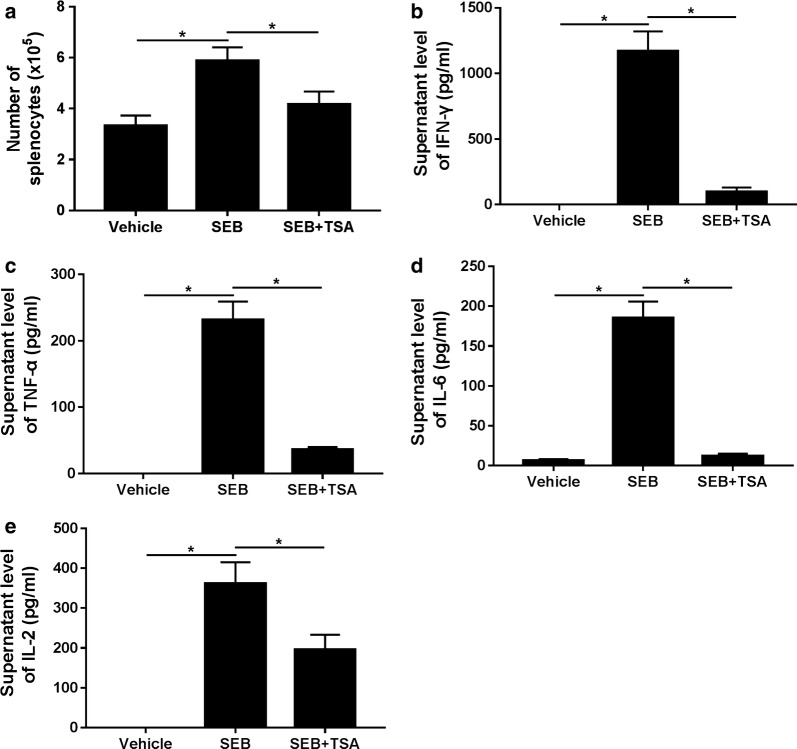
TSA alleviated SEB-induced activation and pro-inflammatory cytokine release of splenocytes. **a** The number of treated splenocytes was analyzed by flow cytometry analysis. **b** The protein expressions of IFN-γ, TNF-α **c**, IL-6 **d** and IL-2 **e** in the supernatants of the treated splenocytes were measured by ELISA. *P < 0.05. SEB group versus Vehicle or SEB + TSA group, respectively

Splenocytes were treated with SEB, in the absence or presence of 100 nM TSA for 24 and we further explored the effects of TSA on SEB-induced ALI in vitro. As shown in Fig. 5a, SEB exposure triggered a substantial increase of the number of splenocytes relative to Vehicle group, which was effectively reduced following the addition of TSA. Additionally, ELISA analysis revealed that the concentrations of IFN-γ (Fig. 5b), TNF-α (Fig. 5c), IL-6 (Fig. 5d) and IL-2 (Fig. 5e) in the supernatants of SEB-stimulated splenocytes were all robustly increased, while these effects were apparently abolished by the administration of TSA. Therefore, these data revealed that TSA alleviated SEB-induced activation of splenocytes and pro-inflammatory cytokine release in splenocytes.

## Discussion

ALI is recognized as a higher destructive disorder that threatens human beings, which is characterized by severe liver inflammation including increased pro-inflammatory cytokine release. Inflammation is well-known to play a relevant role in the pathogenesis of acute and chronic liver injury (Marra and Lotersztajn 2013). Drugs, hepatitis virus, and bacterial toxins including SEB are major causative agents associated with ALI (Krenkel and Tacke 2017). For example, SEB induced lethal toxicity associated with liver injury in endotoxin-resistant C3H/HeJ mice (Yasuda et al. 2002). In the present study, we explored the effects of BBR on SEB-induced ALI. We provided the first evidence that intragastric administration of BBR remarkably attenuated SEB-induced ALI in mice, as demonstrated by the reduction in AST and ALT levels, cellular infiltration of immune cells into the liver and pro-inflammatory cytokine release. Moreover, BBR suppressed activation of splenocytes and pro-inflammatory cytokine release in SEB-stimulated splenocytes. Mechanistic analyses demonstrated that BBR alleviated SEB-mediated ALI via repressing class I HDAC expression, but not class II HDAC.

More recently, many studies have reported the hepatoprotective effect of BBR. For example, it was reported that BBR inhibited carbon tetrachloride-induced liver injury but BBR alone showed little effect on function of liver (Li et al. 2014). BBR could protect against cyclophosphamide-induced hepatotoxicity in rats through attenuating oxidative stress and inflammation (Mahmoud et al. 2014). Additionally, BBR was revealed to protect against methotrexate (MTX)-induced liver injury through upregulating nuclear factor (erythroid-derived 2)-like 2 (Nrf2)/heme oxygenase-1 (HO-1) signaling and peroxisome proliferator activated receptor gamma (PPARγ), and suppressing oxidative stress and apoptosis in rats (Mahmoud et al. 2017). Moreover, BBR was demonstrated to protect ALI in mice through inhibiting inflammation and mitochondria-dependent apoptosis (Xu et al. 2018). Furthermore, BBR was found to ameliorate schistosoma mansoni-induced liver damage and oxidative stress conditions in mice (Dkhil 2014). In our study, we demonstrated that intragastric administration of BBR abated SEB-induced increase of AST and ALT levels, massive cellular infiltration of immune cells into the liver and pro-inflammatory cytokine release in d-gal-sensitized mice, suggesting that BBR remarkably attenuated SEB-induced ALI in mice. We hypothesized whether immune cells were required for BBR-mediated effect in ALI. Hence, splenocytes were isolated and used for further study. We found that BBR treatment counteracted SEB-induced elevation of the number of splenocytes and production of pro-inflammatory cytokines, indicating that BBR alleviated SEB-induced ALI in vitro. Similarly, our previous study proofed that natural indoles, indole-3-carbinol (I3C) and 3,3′-diindolylmethane (DIM), attenuated SEB-induced acute hepatic injury, as evidenced by decrease in AST levels, inflammatory cytokines and cellular infiltration in the liver (Busbee et al. 2015).

HDACs have been shown to play a crucial role in the promotion of activation and pro-inflammatory cytokine release following SEB stimulation (Busbee et al. 2014). Increasing evidence has suggested that small molecule inhibitors of HDAC, such as TSA, exert anti-inflammatory effects in multiple inflammatory models by several mechanisms. For example, it was documented that TSA improved the hepatic injury by inhibiting IL-6 expression in septic mice (Zhang et al. 2009). Besides, TSA alleviated the lesion in liver as well as in small intestine and colon in acute liver failure rats by inflammatory inhibition (Zhang et al. 2016). In the present study, we discovered that 1 μM or 2 μM BBR and TSA both weakened SEB-induced enhancement of HDAC activity in splenocytes. Additionally, qRT-PCR analyses proved that SEB stimulation activated class I HDAC enzyme but suppressed class II HDAC enzyme, while 1 μM or 2 μM BBR and TSA dramatically suppressed SEB-induced activation of class I HDAC enzyme. It is interesting to note that 1 μM or 2 μM BBR and TSA treatment led to a marked elevation of the protein level of H3K9Ac in SEB-stimulated splenocytes, suggesting that BBR was effective at downregulating class I HDAC after activation of SEB. Moreover, we further discovered that TSA alleviated activation of splenocytes and pro-inflammatory cytokine release in SEB-stimulated splenocytes. We inferred from these effects that BBR attenuated SEB-induced ALI through repressing class I HDAC enzyme, but not class II. Similarly, a previous study reported that I3C and DIM repressed SEB-induced T cell activation and cytokine production through acting as class I HDAC inhibitors, but not class II (Busbee et al. 2014).

In summary, our study provided the first evidence that BBR could effectively alleviate SEB-induced ALI and this may be mediated through repressing class I HDAC enzyme. Our study suggested that BBR may constitute a novel therapeutic modality to prevent SEB-mediated inflammation and ALI.

## Abbreviations

SEB: staphylococcal enterotoxin B
ALI: acute liver injury
d-gal: d-galactosamine
ALT: alanine aminotransferase
AST: aspartate aminotransferase
TSA: trichostatin A
*S. aureus*: *Staphylococcus aureus*
MHC II: major histocompatibility complex class II
APCs: antigen-presenting cells
TCR: T cell receptor
TNF-a: tumor necrosis factor-a
IFN-g: interferon-g
HDACs: histone deacetylases
FITC: fluorescein isothiocyanate
ELISA: enzyme-linked immunosorbent assay
